# Endoscopic and rectal tube management of pediatric sigmoid volvulus: A case series

**DOI:** 10.1002/jpr3.12165

**Published:** 2025-02-03

**Authors:** Sapna Khemka, Micah Morris, Kevin Watson

**Affiliations:** ^1^ Akron Children's Hospital Akron Ohio USA; ^2^ Nationwide Children's Hospital Columbus Ohio USA

**Keywords:** endoscopy, pediatric gastroenterology, rectal decompression tube

## Abstract

The process of the sigmoid colon twisting on its mesentery is known as sigmoid volvulus, a diagnosis rarely seen in the pediatric population. Volvulus can lead to blood flow obstruction of the colon and eventually perforation, necrosis, or sepsis. Predisposing factors include chronic constipation, chronic dysmotility, or Hirschsprung disease. This communication demonstrates three patients who presented to a tertiary pediatric care center with a diagnosis of sigmoid volvulus. All three patients underwent immediate endoscopic detorsion and rectal decompression tube placement. Two patients subsequently underwent surgical resection of redundant sigmoid colon. This communication highlights the use of sigmoidoscopy for detorsion of uncomplicated sigmoid volvulus with added support for initial rectal decompression tube placement, contributing to initial patient stabilization and positive patient outcomes.

## Abstract (highlights)

**What is Known:**

- Sigmoid volvulus is a rare pediatric diagnosis consisting of the sigmoid colon twisting on its mesentery.
- Immediate intervention of sigmoid volvulus is required to prevent bowel perforation, necrosis, or sepsis.
- Diagnostic imaging includes abdominal x‐ray or computed tomography.

**What is New:**

- Management of sigmoid volvulus may consist of immediate endoscopic detorsion with colonoscopy and subsequent surgical intervention, if no other contraindications such as bowel necrosis/gangrene, perforation, or peritonitis already present.
- There is added support for use of rectal decompression tube in the acute setting with endoscopic detorsion to prevent recurrence and contribute to initial patient stabilization.

## 1 INTRODUCTION

Sigmoid volvulus occurs when the redundant sigmoid colon twists on its narrow and elongated mesentery, leading to vascular obstruction and closed loop distention.^1^, ^2^ Predisposing factors include older age, chronic constipation, dysmotility, especially in the setting of neurodevelopmental disorders, Hirschsprung disease (HD), and roundworm infection.^1^, ^3^, ^4^, ^5^ Patients can present with abdominal pain, distension, constipation, obstipation, nausea, and vomiting.^1^ Laboratory workup is often unremarkable but can exhibit leukocytosis, while abdominal imaging typically demonstrates dilated colon proximal to obstruction.^1^, ^3^ Diagnosis is considered a medical emergency, and patients may require endoscopic detorsion followed by colonic resection to prevent recurrence.^1^, ^3^ Without urgent intervention, the volvulus can lead to perforation, necrosis, or sepsis due to vascular infarction.^3^

## 2 CASE SERIES

We present three patients diagnosed with sigmoid volvulus on imaging at a pediatric tertiary care center, prompting sigmoidoscopic detorsion with rectal decompression tube placement. Management provided prompt relief of symptoms without immediate recurrence of sigmoid volvulus.

**Figure 1:**
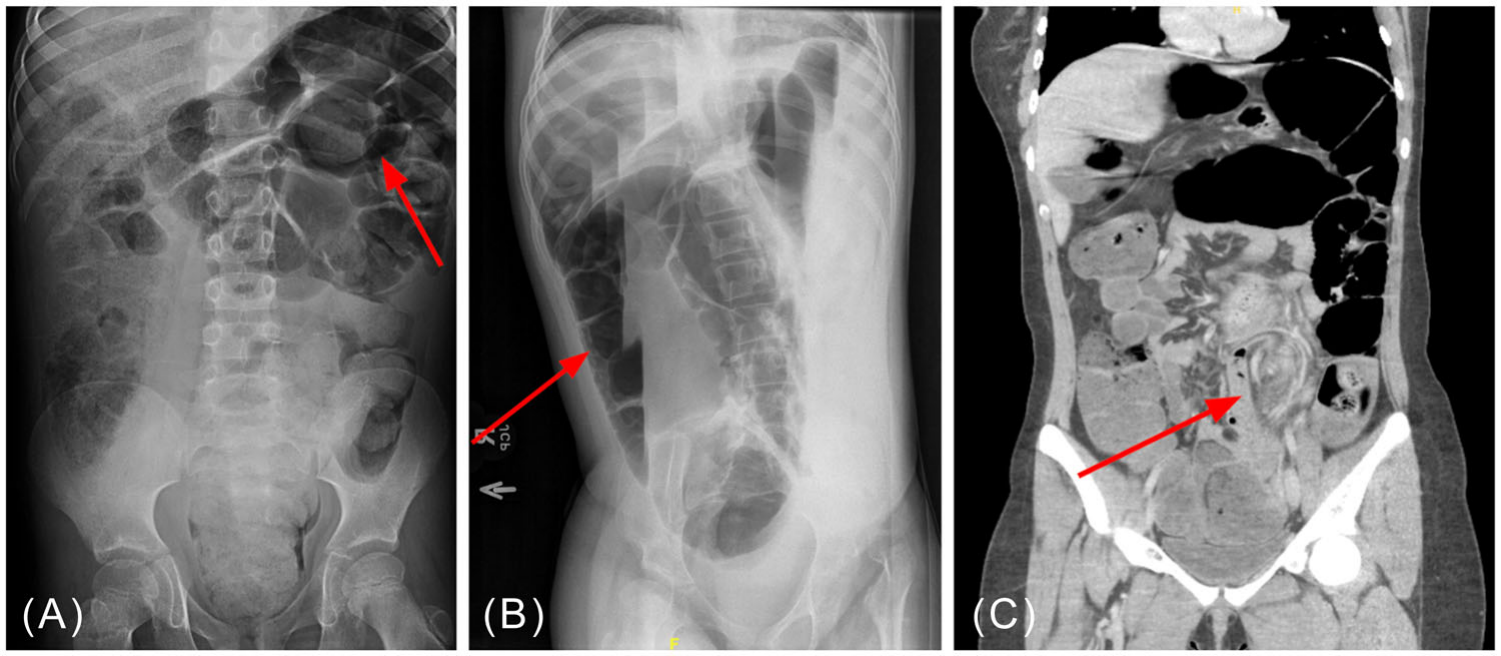
Radiographic images demonstrating classic signs seen in sigmoid volvulus. (A) Patient A's abdominal x‐ray demonstrating dilated colon. (B) Patient B's left lateral decubitus x‐ray demonstrating “coffee bean sign.” (C) Patient C's abdominal computed tomography demonstrating “whirl sign.”

Patient A, an 8‐year‐old male with developmental delay and chronic constipation, presented in January 2018 with 2 days of emesis, abdominal pain, and abdominal distension concerning for bowel obstruction. The previous HD evaluation was negative. At the outside emergency department (ED), he received Fleet® enema and produced two large bowel movements before any diagnostic imaging. Upon transfer to a tertiary care center, an abdominal x‐ray demonstrated colonic distension consistent with sigmoid volvulus (Figure 1A). The next day, Patient A underwent endoscopic detorsion with rectal decompression tube placement. After 2 days, he returned to the operating room (OR) for redundant sigmoid colon resection with end‐end anastomosis. Patient A was discharged home 7 days after detorsion and 5 days after surgical resection with no recurrence of volvulus after initial presentation.

Patient B, an 11‐year‐old female with spastic tetraplegic cerebral palsy and gastrostomy‐jejunostomy (GJ) tube feed dependence, presented in March 2017 with 3 days of constipation, abdominal distension, and feeding intolerance to a routine outpatient pulmonology appointment. Chest x‐ray was obtained for evaluation of chronic respiratory failure where colonic gaseous distension was noted. Patient B had not previously undergone HD evaluation. Subsequent abdominal x‐ray demonstrated colonic dilation measuring 12 cm in diameter with air in the nondependent side on the left lateral decubitus film, concerning for sigmoid volvulus (Figure 1B). The patient was immediately taken to the OR for colonoscopic detorsion. The sigmoid colon was redundant and dilated despite the absence of converging colonic mucosa (whirl sign), prompting rectal decompression tube placement. Four days after endoscopic detorsion, Patient B tolerated feeds and was discharged home. This patient did not undergo interval sigmoidectomy due to diagnostic uncertainty of volvulus versus ileus in the setting of chronic medical illness. She has had no recurrence of volvulus but has had further episodes of ileus.

Patient C, a 16‐year‐old female with history of abdominal migraines, presented with 4 days of abdominal pain, constipation, and worsening abdominal distention in January 2023. An abdominal computed tomography (CT) scan performed at the referring ED demonstrated concern for sigmoid volvulus (Figure 1C), prompting transfer to the tertiary care center. She successfully underwent endoscopic detorsion and rectal decompression tube placement the same day. Tubing was displaced with bowel movement 3 days after placement. Patient C was discharged home with no acute issues. Surgical intervention was postponed due to the initial severity of Patient C's symptoms and marked improvement after detorsion; instead, proceeding with an elective surgery after discharge. Patient C did not have any volvulus recurrence before undergoing sigmoidectomy of redundant colon with end‐end anastomosis 2 months after detorsion. Rectal biopsy at the time was negative for HD. Patient C has had no recurrence of volvulus.

## 3 DISCUSSION

Volvulus in the pediatric population is often isolated in the small bowel due to association with malrotation or internal hernia, making sigmoid volvulus a rare pediatric diagnosis.^2^, ^6^ Damkjaer et al. reports only 100 pediatric cases of sigmoid volvulus from 1940 to 2021.^6^ Common associations with pediatric sigmoid volvulus include chronic constipation and neuropsychiatric disorders. Other predisposing factors include a high‐fiber diet, dysmotility, pregnancy, and HD.^2^ In this case series, Patient A had a history of chronic constipation, which most likely contributed to his episode of sigmoid volvulus. Patient B may have had underlying dysmotility requiring GJ tube feedings. Patient C did not have a notable history of increased risk of sigmoid volvulus. Previous studies demonstrate an incidence of HD in up to 18% of patients with sigmoid volvulus, making it imperative that this etiology is ruled out during evaluation to prevent recurrence and further complications such as constipation.^3^ Evaluation for HD was performed in both Patients A and C, whereas Patient B had not undergone evaluation.

Imaging can be useful in the diagnosis of sigmoid volvulus when patients present with relevant symptoms, including abdominal pain, constipation, and/or emesis. Abdominal x‐ray is the first choice imaging modality.^7^ Classic signs of sigmoid volvulus include a dilated colon with a “U shape,” also known as a coffee bean sign or a bird beak appearance such as that seen in Figure 1A,B.^6^ The addition of barium enema increases radiographic diagnostic sensitivity when abdominal x‐rays are nonspecific for sigmoid volvulus in pediatrics.^6^, ^7^ An abdominal CT scan may be performed if x‐ray is inconclusive despite a high index of suspicion. Abdominal CT imaging would be expected to demonstrate the rotation of the colonic mesentery represented by a “whirl sign,” as seen in Figure 1C.^6^ Inflammation of the colonic wall, distension of the sigmoid colon, and bowel obstruction with proximal dilation may also represent sigmoid volvulus on imaging.^6^

While most literature describes the treatment of sigmoid volvulus to be in the adult population given larger diagnostic and treatment incidence, a systematic review conducted by Parolini et al. describes initial management of sigmoid volvulus in childhood to include water contrast enema along with endoscopic detorsion and decompression if no other contraindications such as bowel necrosis, gangrene, perforation, or peritonitis are already present.^3^, ^4^, ^5^ After initial detorsion, rectal decompression tube placement remains a topic of discussion for mainstay treatment to prevent early relapse of volvulus, while definitive treatment has been demonstrated by colonic resection.^2^ Damkjaer et al. has demonstrated up to 35% recurrence of volvulus without sigmoidectomy.^6^ There has also been increased recurrence noted in patients who have spontaneous decompression of sigmoid volvulus, which Aksunger et al. describe as up to 2% of cases worldwide.^8^ These data make Patient B's presentation possibly a spontaneously reduced sigmoid volvulus or, more likely, an ileus versus a sigmoid volvulus despite initial imaging given the absence of recurrence.

**Figure 2:**
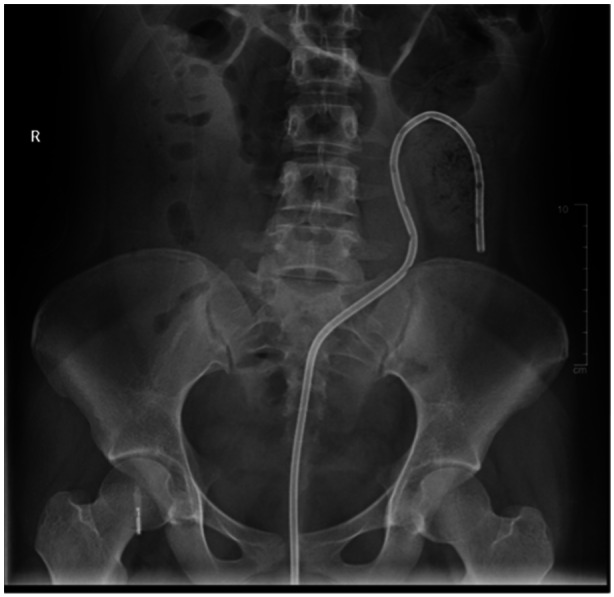
Patient C's abdominal x‐ray demonstrating rectal decompression tube catheter after placement.

Pediatric patients require general anesthesia while undergoing colonic detorsion in the OR. As the endoscope enters the colon, a whirl sign may be present at the recto‐sigmoid junction indicating the distal end of the sigmoid volvulus.^3^, ^9^ The endoscope is advanced into the dilated colon and immediate suctioning is performed as the scope is torqued clockwise.^9^ The endoscope is further advanced to the proximal point of the volvulus, where additional suctioning is performed.^9^ Once detorsion and initial decompression of the dilated sigmoid loop have been completed, a rectal decompression tube may be placed to provide patient stabilization and prevent early volvulus relapse.^3^, ^10^, ^11^ A guidewire is inserted through the endoscopic channel and advanced into the descending colon as the endoscope is withdrawn. The rectal tube catheter is placed over the guidewire and advanced under fluoroscopy past the point of colonic obstruction (Figure 2). The guidewire is removed, and the rectal tube is secured via tape to the perianal skin and left in place for several days.^9^ The endoscope may be reintroduced to secure tubing to the colonic mucosa with endoscopic clip before surgical resection.^3^, ^10^ The goals of endoscopic detorsion with the placement of rectal decompression tube focus on relieving the obstruction while preventing bowel ischemia and recurrence.^9^ The complications that may occur with colonic detorsion include the risk of bleeding, infection, and perforation. These risks are increased in an emergent surgical intervention setting compared to an elective operation.^9^ Broad‐spectrum antibiotic administration is only necessary in the setting of concern for peritonitis, sepsis, or ischemia.^9^ Another risk that must be considered is the inability to endoscopically reduce the torsion, requiring further and emergent surgical intervention.

Although sigmoid volvulus is rarely seen in the pediatric population, a high index of suspicion allows for early detection and intervention, preventing the development of consequences including perforation, necrosis, and septic shock.^2^, ^3^ All three patients described successfully underwent endoscopic detorsion within 24 h of presentation, allowing for early intervention and reduced risk of morbidity. This case series suggests that sigmoidoscopy may be an effective approach for detorsion of uncomplicated pediatric sigmoid volvulus. It also highlights the use of rectal decompression tubes, as none of the patients experienced acute recurrence during hospitalization, contributing to initial patient stabilization and positive patient outcomes.

## CONFLICT OF INTEREST STATEMENT

The authors declare no conflicts of interest.

## ETHICS STATEMENT

This case series was reviewed and deemed exempt from institutional review board oversight. Informed consent was obtained from the parents/guardians of all patients for the publication of case details.
